# A Comprehensive RNA Expression Signature for Cervical Squamous Cell Carcinoma Prognosis

**DOI:** 10.3389/fgene.2018.00696

**Published:** 2019-01-04

**Authors:** Jie Xiong, Shengyu Guo, Zhitong Bing, Yanlin Su, Le Guo

**Affiliations:** ^1^Department of Epidemiology and Health Statistics, Xiangya School of Public Health, Central South University, Changsha, China; ^2^Department of Computational Physics, Institute of Modern Physics of Chinese Academy of Sciences, Lanzhou, China; ^3^Department of Gynaecology and Obstetrics, Changsha Central Hospital, Changsha, China; ^4^The First Department of Operation, Hunan Provincial People’s Hospital, Changsha, China

**Keywords:** transcriptome, signature, prognosis, CESC, angiogenesis

## Abstract

Clinicopathological characteristics alone are not enough to predict the survival of patients with cervical squamous cell carcinoma (CESC) due to clinical heterogeneity. In recent years, many genes and non-coding RNAs have been shown to be oncogenes or tumor-suppressors in CESC cells. This study aimed to develop a comprehensive transcriptomic signature for CESC patient prognosis. Univariate, multivariate, and Least Absolute Shrinkage and Selection Operator penalized Cox regression were used to identify prognostic signatures for CESC patients from transcriptomic data of The Cancer Genome Atlas. A normalized prognostic index (NPI) was formulated as a synthetical index for CESC prognosis. Time-dependent receiver operating characteristic curve analysis was used to compare prognostic signatures. A prognostic transcriptomic signature was identified, including 1 microRNA, 1 long non-coding RNA, and 6 messenger RNAs. Decreased survival was associated with CESC patients being in the high-risk group stratified by NPI. The NPI was an independent predictor for CESC patient prognosis and it outperformed the known clinicopathological characteristics, microRNA-only signature, gene-only signature, and previously identified microRNA and gene signatures. Function and pathway enrichment analysis revealed that the identified prognostic RNAs were mainly involved in angiogenesis. In conclusion, we proposed a transcriptomic signature for CESC prognosis and it may be useful for effective clinical risk management of CESC patients. Moreover, RNAs in the transcriptomic signature provided clues for downstream experimental validation and mechanism exploration.

## Introduction

Cervical cancer (CC) is still the fourth most common cancer in women (Ferlay et al., 2015). Despite developed countries are low epidemic areas of CC by virtue of easier accessibility of routine screening test and human papillomavirus (HPV) vaccination, CC is still the second leading cause of cancer death among women aged 20–39 years in the United States in 2015 (Siegel et al., 2018). At present, clinical stage is the leading predictive characteristic for CC prognosis, although useful, significant variability is observed and the 5 years survival rate is still poor for women with advanced CC (30–40% for stage III and 15% for stage IV). Theoretically, clinicopathological characteristics are macroscopic emergence of molecules (e.g., genes, proteins) and CC patients with homogeneous clinical status may have completely diverse molecular patterns. Therefore, identification of robust and accurate molecular biomarkers for CC patient prognosis is valuable and in urgent need.

By comprehensively characterizing various molecules (DNA-level, RNA-level, protein-level) in 100s of CC samples, The Cancer Genome Atlas (TCGA) has provided a comprehensive way to understand CC (The Cancer Genome Atlas Research Network et al., 2017). Enormous multiple omics data make the discovery of potential biomarkers for CC diagnosis, treatment and prognosis possible. Several studies have investigated the molecular signatures for CC prognosis based on the expression of CC genome. Hu et al. (2010) profiled 96 cancer-related microRNAs (miRNAs) in 102 CC samples and firstly proposed a two-miRNA expression signature for predicting the overall survival (OS) of CC patients. How et al. (2015) measured the miRNA omics of CC samples by miRNA arrays and proposed a prognostic nine-miRNA expression signature in their training set. However, the prognostic value of the nine-miRNA expression signature could not be validated in an independent cohort (How et al., 2015). Liu et al. (2016), Liang et al. (2017), Ma et al. (2018), and Ying et al. (2018) proposed a seven-miRNA expression signature, a three-miRNA expression signature, a three-miRNA expression signature, and a 2 two-miRNA expression signature for CC prognosis based on TCGA miRNA sequencing data, respectively. Huang et al. (2012) profiled 1440 human tumor related gene transcripts using custom oligonucleotide microarrays in 100 CC samples and identified a prognostic seven-gene expression signature. Based on TCGA gene sequencing data, Li et al. (2017b, 2018) proposed a two-histone family gene signature and further proposed another independent gene signature to predict the OS of CC patients.

However, some limitations should be noticed: (1) Previous studies focused on single omics independently, and there lacks a whole transcriptomic analysis which may provide more comprehensive and robust discovery (Hu et al., 2010; Huang et al., 2012; How et al., 2015; Liu et al., 2016; Li et al., 2017b, 2018; Liang et al., 2017; Ma et al., 2018; Ying et al., 2018). (2) Prognostic miRNA signatures identified based on the same data source without cross-references are very different (Liu et al., 2016; Liang et al., 2017; Ma et al., 2018; Ying et al., 2018). (3) For prognostic miRNAs, previous studies did not distinguish miRNA isoforms (3p-arm or 5p-arm). Thus, it is unclear which isoform should be further investigated by experiment (Hu et al., 2010; How et al., 2015; Liu et al., 2016; Liang et al., 2017; Ma et al., 2018; Ying et al., 2018). (4) Pathologically, CC includes cervical squamous cell carcinoma (CESC) and cervical adenocarcinoma (CADC). Because there are significant differences in prognosis between CESC and CADC (Jung et al., 2017), it is not appropriate to mix them for identification of prognostic biomarkers. (5) Semi-parametric survival analysis method such as Cox regression analysis is loosely used without checking the proportional hazards (PH) assumption (Hu et al., 2010; Huang et al., 2012; How et al., 2015; Liu et al., 2016; Li et al., 2017b, 2018; Liang et al., 2017; Ma et al., 2018; Ying et al., 2018). (6) To identify prognostic signatures from high-dimensional omics data, classical multivariate Cox regression analysis (MCA) is usually impeded by the “curse of dimensionality” (i.e., low sample size and large number of variables), which leads to over-fitting and unstable estimation of regression coefficients.

To address these limitations, we submit the transcriptomic data of CESC patients to a Least Absolute Shrinkage and Selection Operator (LASSO) penalized MCA (Simon et al., 2011) to identify a transcriptomic signature for CESC prognosis.

## Materials and Methods

### Data Acquisition

Level 1 clinical data, level 3 transcriptomic sequencing data, and the corresponding metadata of CCs were retrieved and downloaded from TCGA^1^ repository in January 2018. Search strategies can be obtained in Section I of the Supplementary Material. CC patients were included in this study by the following criteria: (1) CC patients diagnosed as CESC; (2) CESC patients with at least 60 days follow-up; (3) CESC patients have both clinical data, gene sequencing data, and miRNA isoform sequencing data; (4) CESC patients do not have prior other malignancies; and (5) CESC patients do not receive any preoperative neoadjuvant therapy.

### Data Preprocessing

Clinical eXtemsible Markup Language files were parsed by R “XML” package and the R code can be achieved in Section II of the Supplementary Material. Details on sequencing data preprocessing can be also available in Section II of the Supplementary Material. Hierarchical clustering was used to cluster samples to detect sample outliers and guided principal component analysis (gPCA) (Reese et al., 2013) was adopted to evaluate batch effects of the sequencing data.

### Identification of Prognostic Demographic and Clinicopathological Characteristics

Kaplan–Meier (KM) survival analysis with log-rank test was applied to evaluate the prognostic effects of age at diagnosis, clinical stage, menopause status, ethnicity, birth control pill usage, tobacco usage, and lymphovascular invasion for CESC. Furthermore, MCA with demographic and clinicopathological characteristics as covariates was adopted to evaluate their independence for CESC prognosis.

### Univariate Survival Analysis of RNAs

The miRNA isoform sequencing data only include miRNAs while the gene sequencing data include both messenger RNAs (mRNAs) and long non-coding RNAs (lncRNAs). For convenience of description, we termed mRNA and lncRNA as gene and further termed miRNA, mRNA and lncRNA as RNA.

Associations between OS and RNA expression profiles were preliminarily evaluated by univariate Cox regression analysis (UCA). The proportional hazards (PH) assumption was tested by Schoenfeld residual (Grambsch and Therneau, 1994) and a unified multiple testing (Strimmer, 2008) was applied for tail area-based false discovery rate (FDR) estimation. RNAs with FDRs < 0.1 and PH assumption test P values >0.1 (Kleinbaum and Klein, 2012) were considered to be preliminarily associated with OS of CESC patients. Furthermore, RNAs with hazard ratios (HRs) > 1 were defined to be risky for CESC prognosis, and those with HRs < 1 were considered as protective.

### Multivariate Analysis of Preliminarily Survival Associated RNAs

For miRNAs, stepwise MCA was applied to preliminarily survival associated miRNAs to construct an independent miRNA-only expression signature for CESC prognosis. The Bayesian information criterion (BIC) (Schwarz, 1978) was adopted for model selection.

Because the number of preliminarily survival associated genes was comparable to the number of samples, a LASSO penalized MCA with 10-fold cross validation and 1000 iterations was adopted to select genes by penalizing low regression coefficients exactly to zero. To alleviate the local minimum problem, we repeated the LASSO penalized MCA 10 times with different initializations and the model that achieved the minimal partial likelihood was adopted. Furthermore, stepwise MCA was applied to genes selected by the LASSO penalized MCA to construct an independent gene-only expression signature for CESC prognosis.

Finally, a transcriptomic signature for CESC prognosis was constructed by stepwise MCA with gene-only signature and miRNA-only signature as covariates.

### Risk Score

A normalized prognosis index (NPI) defined as the standard form of a linear combination of the observed values weighted by the regression coefficients was adopted as a synthetical index for CESC prognosis. Specifically,

$$\mathrm{NPI}=\frac{\mathrm{PI}-\mathrm{mean}(\mathrm{PI})}{\mathrm{sd}(\mathrm{PI})},$$

where PI is a prognostic index vector and the *j*th element of PI is the prognostic index of the *j*th patient, i.e.

$${\mathrm{PI}}_{j}=\sum_{i}\beta_{i}\times G_{\mathrm{ij}},$$

β_*i*_ is the regression coefficient of the *i*th variable (in this context, the *i*th gene/miRNA); *G_ij_* is the observed value of the *i*th variable in the *j*th sample (in this context, the expression of the *i*th gene/miRNA in the *j*th sample). For miRNA-only signature, gene-only signature, the integrated RNA signature (i.e., transcriptomic signature), and the previously identified prognostic signatures, we termed the corresponding NPI as miRNA-NPI, gene-NPI, RNA-NPI, and pre-NPI, respectively.

### Model Evaluation and Comparison

CESC patients were stratified into a high-risk group (NPI > 0) or a low-risk group (NPI < 0) based on NPI. OS between the high-risk group and the low-risk group was compared by KM survival analysis. MCA was used to evaluate the independence of various NPIs and clinical factors. The abilities of various NPIs to predict CESC patient survival outcome were assessed and compared by calculating the area under the curve (AUC) of the time-dependent receiver operating characteristic (ROC) at 3, 5, and 10 years, respectively.

### Gene Ontology and Pathway Enrichment

Unlike other mRNA target prediction software just based on sequence alignment, miRTarBase provided experimentally validated mRNA targets of miRNA. Both strongly and weakly validated mRNA targets of the prognostic miRNA were obtained from miRTarBase (version 7.0) (Chou et al., 2018). Metascapae (Tripathi et al., 2015)^2^ was adopted for gene ontology and pathway enrichment of the prognostic mRNAs and targets of the prognostic miRNA.

### Statistical Analysis Tools

*P*-value less than 0.05 or adjusted *P*-value less than 0.1 was considered to be significant. All analyses were performed by R software. Non-parametric survival analysis, semi-parametric survival analysis, and PH assumption test were performed by *survival* and *survminer* packages. LASSO penalized MCA was conducted by textitglmnet package. Multiple test correction was conducted by *fdrtool* package. Time-dependent ROC analysis was conducted by *timeROC* package.

## Results

### Available Data

**FIGURE 1:**
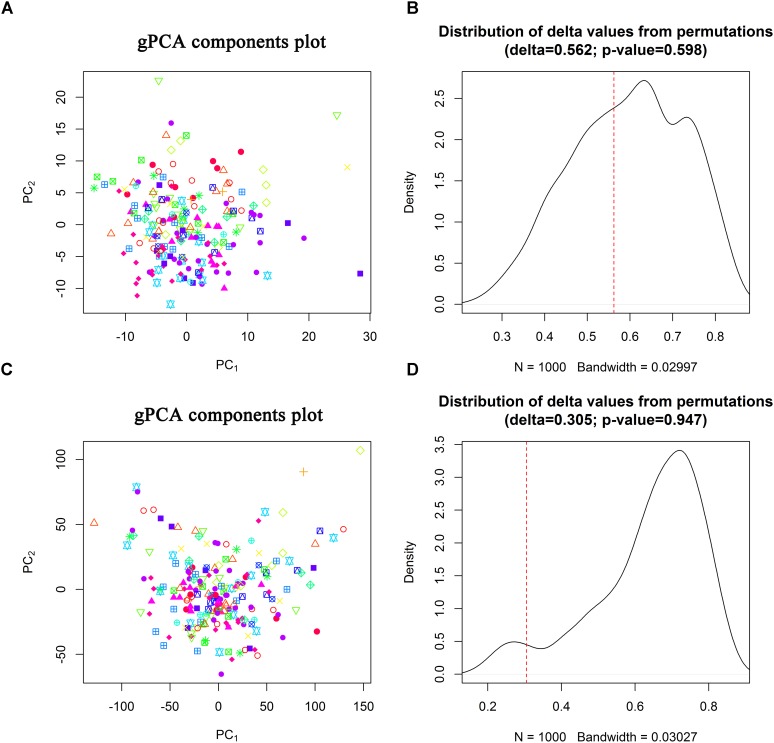
Batch effect evaluation. Scatter plots of the first two guided principal components **(A,C)** and permutation tests **(B,D)** for miRNA isofrom sequencing data and gene sequencing data.

TCGA CC dataset included 307 CC patients who had generated 312 samples for miRNA sequencing (including 307 primary CC samples, 2 metastatic CC samples, and 3 normal samples) and 309 samples for gene sequencing (including 304 primary CC samples, 2 metastatic CC samples, and 3 normal samples). Due to small number of metastatic and normal CC samples, we only analyzed the primary CC samples. Based on the inclusion criteria and low-expressed RNA filtering (Supplementary Material Section III), 214 CESC samples covering 401 miRNAs and 13631 genes (mRNAs and lncRNAs) were retained. Hierarchical clustering showed that there existed a miRNA sample outlier and seven gene sample outliers. After removing sample outliers and scaling the expressions of miRNAs and genes to zero sample mean and standard deviation, 206 primary CESC samples were included for identification of prognostic signatures. Batch effect analysis showed that there was no obvious separation on the first two guided principal components for both miRNA isoform sequencing data and gene sequencing data (Figures 1A,C) with permutation test *P*-values of 0.598 and 0.947 (Figures 1B,D), respectively. These results indicated that there was no significant batch effect in the sequencing data.

### Prognostic Demographic and Clinicopathological Characteristics

Of the 206 patients in TCGA-CESC cohort, 55 patients were deceased and 151 were alive at the time of last follow-up. The median OS time was 3097 days (95% CI: 2859–NA days). Demographic and clinicopathological characteristics for TCGA-CESC cohort are summarized in Table 1. Age at initial diagnosis (HR = 1.81, 95% CI: 0.92–3.58), clinical stage (HR = 2.14, 95% CI: 1.12–4.09), tobacco usage (HR = 2.36, 95% CI: 1.24–4.48), and lymphovascular invasion (HR = 13.70, 95% CI: 5.64–33.31) were negatively associated with OS of CESC patients revealed by KM survival analysis (Table 1 and Supplementary Figure S1). MCA revealed that only lymphovascular invasion was an independent predictor for CESC prognosis (Table 1). However, information of lymphovascular invasion was not available in more than half of the CESC patients (*n* = 106). Considering age at initial diagnosis, clinical stage, and tobacco usage as covariates (i.e., without lymphovascular invasion), MCA revealed that only clinical stage was an independent prognostic clinicopathological characteristic (Table 1).

**Table 1:** Patient characteristics, KM survival analysis, and MCA of demographic and clinicopathological features.

Variables	^U^KM survival analysis				^M^HR (95% CI)		^M^P (^M−^P)
	Deaths/Patients (%)	HR	95% CI	Log-rank test P		
**Age at diagnosis^∗^**						
≥60 years	16/39 (41.03)	1.81	0.92–3.58	0.042	1.414 (0.746–2.681)	0.759 (0.289)
<60 years	39/167 (23.35)					
**Clinical stage^∗∗^**						
Stage III–IV	20/48 (41.67)	2.14	1.12–4.09	0.005	1.925 (1.053–3.520)	0.994 (0.033)
Stage I–II	35/153 (22.88)					
**Menopause status^$^**						
Pre	19/87 (21.84)	1.14	0.61–2.13	0.921		
Peri	4/19 (21.05)					
Post	16/58 (27.59)					
**Ethnicity**						
HISPANIC OR LATINO	1/16 (6.25)	0.51	0.12–2.19	0.486		
NOT HISPANIC OR LATINO	28/114 (24.56)					
**Birth control pill usage^$^**						
Current user	2/8 (25.00)	1.44	0.63–3.28	0.625		
Former user	6/38 (15.79)					
Never used	15/62 (24.19)					
**Tobacco usage^∗∗$^**						
Current smoker	22/51 (43.14)	2.36	1.24–4.48	0.005	1.391 (0.997–1.941)	0.504 (0.052)
Reformed smoker	7/36 (19.44)					
Non-smoker	21/102 (20.59)					
**Lymphovascular invasion^∗∗∗^**						
Present	19/58 (32.76)	13.70	5.64–33.31	0.0008	13.954 (1.864–104.4)	0.019
Absent	1/42 (2.38)					

*HR, hazard ratio; CI, confidence interval; ^U^, univariate analysis; ^M^, multivariate analysis; ^M^P, P-value derived from MCA including Age at diagnosis, Clinical stage, tobacco usage, and lymphovascular invasion as covariates; ^M−^P, P-value derived from MCA without lymphovascular invasion. P-values were obtained from log-rank test for KM survival analysis and Wald test for Cox regression, patients were omitted when data is unavailable. For MCA, variable coding is as follows: Age at diagnosis (1 ≤ 60 years; 2 ≥ 60 years), Clinical stage (1 = leve I–II; 2 = level III–IV), tobacco usage (1 = Non smoker; 2 = Reformed smoker; 3 = Current smoker), and lymphovascular invasion (1 = Absent; 2 = Present). Patients with missing values were omitted to display in the table. ^∗^P < 0.05, ^∗∗^P < 0.01, and ^∗∗∗^P < 0.001. ^$^the HR was calculated between the first value and the remaining values for variables that have more than two values.*

### miRNA-Only Expression Signature for CESC Prognosis

**FIGURE 2:**
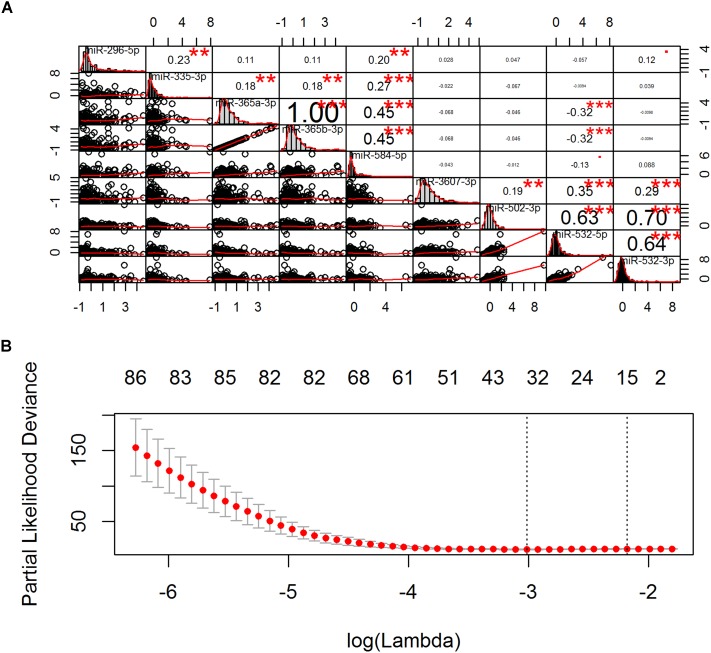
Pairwise correlation and cross-validation. **(A)** The diagonal, upper triangular, and lower triangular of the correlation plot is the histogram, scatter plot, and correlation coefficient and significance, respectively. ^∗^*P* < 0.05, ^∗∗^*P* < 0.01, ^∗∗∗^*P* < 0.001. **(B)** The left vertical line shows where the cross-validation error curve hits its minimum, the right vertical line shows the most regularized model with cross-validation error within 1 standard deviation of the minimum, and the numbers at the top of the Figure indicate the number of the nonzero coefficients. The optimal model is chosen where the cross-validation error curve hits its minimum (left vertical line).

Univariate Cox regression analysis (UCA) after FDR correction (Supplementary Figure S2) revealed that 9 miRNAs were significantly associated with OS of CESC patients. Spearman correlation analysis showed that these prognostic miRNAs were correlated with each other in the upper left block (including hsa-miR-296-5p, hsa-miR-335-3p, hsa-miR-365a-3p, hsa-miR-365b-3p, and hsa-miR-584-5p) and the lower right block (including hsa-miR-3607-3p, hsa-miR-502-3p, hsa-miR-532-5p, and hsa-miR-532-3p) (Figure 2A). To obtain non-redundant miRNAs to predict the OS of CESC patient, stepwise MCA was performed in each block. Hsa-miR-335-3p and hsa-miR-365b-3p were selected by BIC to represent the upper left block and hsa-miR-532-5p was selected to represent the lower right block. Finally, considering hsa-miR-335-3p, hsa-miR-365b-3p, and hsa-miR-532-5p as covariates, MCA reveled that hsa-miR-335-3p and hsa-miR-532-5p were independent prognostic miRNAs and hsa-miR-365b-3p was discarded due to the correlations between blocks (Figure 2A). Furthermore, PH assumption test revealed that hsa-miR-335-3p did not satisfy the PH assumption in the final stepwise Cox model (Supplementary Figure S3). Thus, we obtained hsa-miR-532-5p as an independent miRNA expression signature for CESC prognosis (Table 2).

**Table 2:** miRNA signature, gene signature, and transcriptomic signature.

Signature	Symbol	^M^HR (95% CI)	^M^*P*-value	Type	Model *P*-value
**miRNA**					
MIMAT0002888	hsa-mir-532-5p	0.30 (0.17–0.54)	4.81e-05	Protective	4.81e-05
**Gene**					
ENSG00000132819.15	RBM38	0.42 (0.27–0.65)	1.38e-04	Protective	6.22e-15
ENSG00000081041.8	CXCL2	1.83 (1.50–2.23)	3.12e-09	Risky	
ENSG00000043355.9	ZIC2	0.35 (0.21–0.59)	7.80e-05	Protective	
ENSG00000014914.18	MTMR11	1.46 (1.23–1.74)	2.23e-05	Risky	
ENSG00000170955.9	CAVIN3	1.45 (1.20–1.76)	1.33e-04	Risky	
ENSG00000176124.10	DLEU1	2.08 (1.57–2.75)	3.10e-07	Risky	
ENSG00000135766.8	EGLN1	1.92 (1.55–2.39)	2.65e-09	Risky	
ENSG00000169902.12	TPST1	1.38 (1.17–1.63)	1.29e-04	Risky	
**RNA**					
MIMAT0002888	hsa-mir-532-5p	0.30 (0.15–0.58)	3.83e-04	Protective	2.72e-14
ENSG00000132819.15	RBM38	0.35 (0.21–0.57)	2.31e-05	Protective	
ENSG00000081041.8	CXCL2	1.67 (1.36–2.04)	1.04e-06	Risky	
ENSG00000043355.9	ZIC2	0.31 (0.18–0.53)	2.58e-05	Protective	
ENSG00000014914.18	MTMR11	1.59 (1.31–1.92)	2.26e-06	Risky	
ENSG00000176124.10	DLEU1	2.21 (1.61–3.03)	8.42e-07	Risky	
ENSG00000135766.8	EGLN1	1.79 (1.45–2.21)	4.17e-08	Risky	
ENSG00000169902.12	TPST1	1.33 (1.14–1.57)	4.73e-04	Risky	

*HR, hazard ratio; ^M^, multivariate analysis.*

### Gene-Only Expression Signature for CESC Prognosis

UCA after FDR correction (Supplementary Figure S4) revealed that 218 genes were significantly associated with OS of CESC patients. Because the number of genes was larger than the number of samples, LASSO penalized MCA revealed that 38 genes were with non-zero regression coefficients (Figure 2B). Stepwise MCA further revealed that 1 lncRNA 7 mRNAs were optimal to construct an independent gene expression signature for CESC prognosis (Table 2).

### Transcriptomic Signature for CESC Prognosis

**FIGURE 3:**
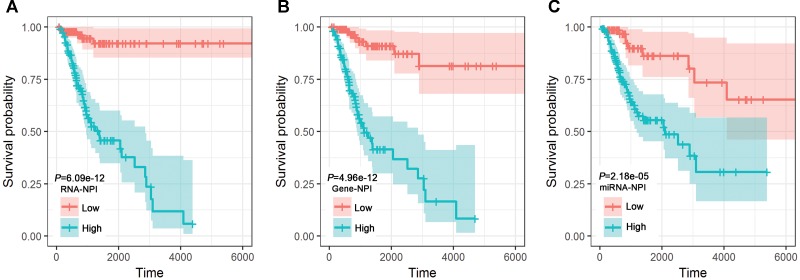
Kaplan-Meier plots for the transcriptomic signature **(A)**, the gene-only signature **(B)**, and the miRNA-only signature **(C)**.

Considering the identified 1 miRNA, 1 lncRNA, and 7 mRNAs as covariates, MCA revealed that hsa-miR-532-5p, lncRNA DLEU1, RBM38, CXCL2, ZIC2, MTMR11, EGLN1, and TPST1 were independent predictors for CESC prognosis (Table 2). NPIs for the miRNA-only signature, the gene-only signature, and the transcriptomic signature were calculated, respectively. Stratification based on RNA-NPI (Figure 3A), gene-NPI (Figure 3B), and miRNA-NPI (Figure 3C) showed that CESC patients in the high-risk group had significantly shorter OS than those in the low-risk group Furthermore, as continuous variables, the miRNA-NPI (HR = 3.32, 95% CI: 1.86–5.93, *P*-value = 4.81E-05), the gene-NPI (HR = 7.89, 95% CI: 5.08-12.25, *P*-value < 2.00E-16), and the RNA-NPI (HR = 13.99, 95% CI: 7.88–24.83, *P*-value < 2.00E-16) were significant predictors for OS of CESC patients revealed by UCA. However, MCA revealed that neither miRNA-NPI (Supplementary Table S1) nor gene-NPI (Supplementary Table S2) was an independent predictor for the OS of CESC patients when considering RNA-NPI as covariate.

To exclude potential effects of mutations on CESC prognosis, we also investigated the mutational patterns of the genes in the identified transcriptomic signature using *OncoPrinter*. Mutations of ZIC2, MTMR11, EGLN1, and TPST1 were found in two, five, one, and two of total 206 CESC samples, and none of these mutations were found in 197 CESC samples. MCA further showed that the identified RNAs were independent predictors for CESC prognosis in the non-mutated samples (Supplementary Table S3). Thus, the prognostic roles of the identified RNAs were merely caused by their expressions.

### Signature Evaluation and Comparison

**FIGURE 4:**
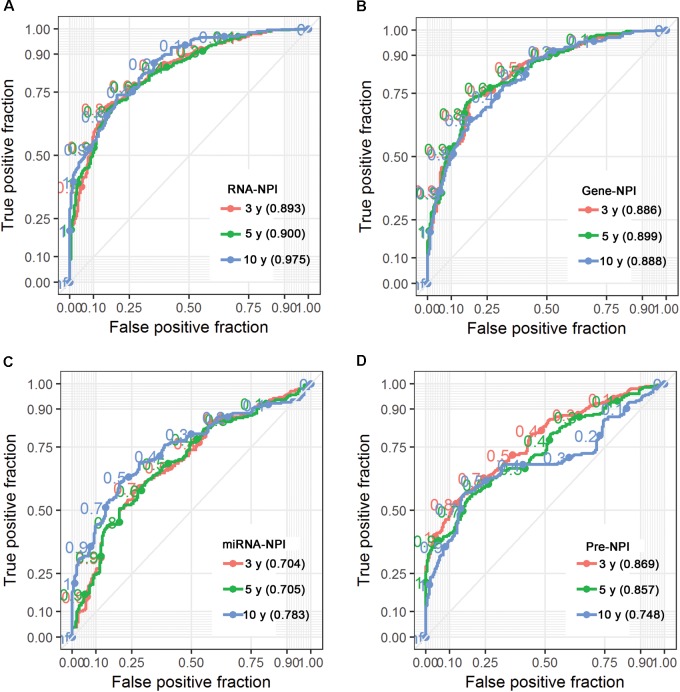
Time dependent ROC for RNA-NPI **(A)**, gene-NPI **(B)**, miRNA-NPI **(C)**, and pre-NPI **(D)**.

Time-dependent ROC analysis showed that the transcriptomic signature (Figure 4A; AUC = 0.893, 0.900, and 0.975 at 3, 5, and 10 years, respectively) had slight better predictive ability for CESC survival than the gene-only signature (Figure 4B; AUC = 0.886, 0.899, and 0.888 at 3, 5, and 10 years, respectively) but better than the miRNA-only signature (Figure 4C; AUC = 0.704, 0.705, 0.783 at 3, 5, and 10 years, respectively).

We also compared the miRNA and gene expression signatures proposed by previous studies (Hu et al., 2010; Huang et al., 2012; How et al., 2015; Liu et al., 2016; Liang et al., 2017; Li et al., 2017b, 2018; Ma et al., 2018; Ying et al., 2018) (Supplementary Table S4) with our transcriptomic signature with respect to independence and predictive ability for CESC prognosis. As shown in Supplementary Table S4, previously proposed miRNA and gene signatures were rarely overlapped across studies except for hsa-miR-378c which was identified by both Liu et al. (2016) and Ma et al. (2018) Specifically, hsa-miR-500a-5p, hsa-miR-500a-3p, hsa-miR-500b-5p, hsa-miR-142-3p, hsa-miR-3607-3p, hsa-miR-502-3p, and hsa-miR-145-5p, RFC4, HIST1H2BD, HIST1H2BJ, and MCM5 could be validated in our study. MCA of these verifiable miRNA and genes found that 1 gene and 3 miRNAs were independent (Supplementary Table S5). We also calculated the NPI for each CESC patient based on the obtained regression model and termed it as pre-NPI. MCA showed that both RNA-NPI and pre-NPI were independent predictors for CESC prognosis (Supplementary Table S6). Finally, time-dependent ROC analysis for pre-NPI (Figure 4D; AUC = 0.869, 0.857, and 0.748 at 3, 5, and 10 years, respectively) demonstrated that the pre-NPI was inferior to the RNA-NPI for prediction of CESC patient survival.

Moreover, MCA revealed that clinical stage, RNA-NPI, and pre-NPI were independent predictors for CESC prognosis (Table 3). However, when considering lymphovascular invasion as covariate, RNA-NPI was the only independent prognostic index (Table 3). These results demonstrated that the transcrip-tomic signature was a better predictor for CESC prognosis compared with clinicopathological characteristics and previous proposed signatures.

**Table 3:** MCA of RNA-NPI, pre-NPI, and clinicopathological features.

	^M−^HR (95% CI)	^M^HR (95% CI)	^M−^P (^M^P)
RNA-NPI	10.34 (5.79–18.46)	18.29 (5.62–59.47)	6.16E-12 (1.36e-06)
pre-NPI	2.51 (1.35–4.64)	2.29 (0.78–6.71)	6.53E-03 (0.132)
Clinical_stage	1.93 (1.09–3.43)	2.17 (0.54–8.64)	4.75E-02 (0.273)
Lymphovascular _invasion	–	6.79 (0.85–54.11)	9.67E-03 (0.007)

*^M^P, P-value derived from multivariate Cox regression analysis including RNA-NPI, pre-NPI, clinical stage, and lymphovascular invasion as covariates; ^M−^P, P-value derived from multivariate Cox regression analysis without lymphovascular invasion.*

### Angiogenesis Related Functions and Pathways

**FIGURE 5:**
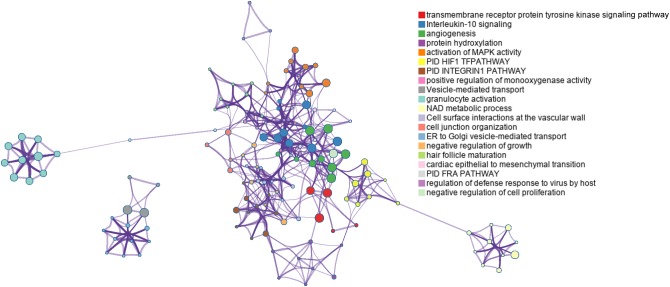
Network of the top 20 enriched gene ontology and pathway terms colored by cluster ID.

Sixty-four genes were validated as targets of hsa-miR-532-5p. Gene ontology and pathway enrichment analyses indicated that targets of hsa-miR-532-5p and the prognostic mRNAs in the transcriptomic signature were mainly associated with angiogenesis (Figure 5). Angiogenesis is a main hallmark of tumor progression and may be an independent prognostic factor in CC (Bremer et al., 1996). In this study, EGLN1 (Egl-9 family hypoxia inducible factor 1; also known as PHD2) is a risky gene (HR = 1.79, 95% CI: 1.45–2.21) and it is closely related with angiogenesis by regulating the stability of HIF1 in non-CESC cancers (Chan and Giaccia, 2010; Lu et al., 2013). Targets of hsa-miR-532-5p such as runt-related transcription factor-3 (Peng et al., 2006; Kim et al., 2016), insulin-like growth factor-binding protein-5 (Rho et al., 2008; Lee et al., 2016), and von Hippel-Lindau (Kong et al., 2011, 2014; Lu et al., 2013) were proved to be associated with angiogenesis in many types of cancer. These results prompted that further experiments aimed to explore functional mechanisms of the transcriptomic signature could be focused on angiogenesis related pathways.

## Discussion

In this study, a novel transcriptomic signature for CESC patient prognosis was identified. Our proposed transcriptomic signature includes two non-coding RNAs (hsa-miR-532-5p and lncRNA DLEU1) and six mRNAs (RBM38, CXCL2, ZIC2, MTMR11, EGLN1, and TPST1). It is natural to wonder if there are any mRNA targets of the non-coding RNAs in the transcriptomic signature. Interestingly, CXCL2 was reported to be a direct target of hsa-miR-532-5p in hepatocellular carcinoma and this miRNA-gene interaction inhibited hepatocellular carcinoma cell proliferation and metastasis (Song et al., 2015). However, correlation analysis revealed that no significant correlation between hsa-miR-532-5p and CXCL2 was observed. Forty-six genes were predicted to be targets of lncRNA-DLEU1 by starBase v2.0 (Li et al., 2014), but none of them were in the transcriptomic signature. For identification of prognostic biomarkers, it is expected to construct signatures that include as many independent biomarkers as possible. Due to the independence among the biomarkers in the transcriptomic signature, it is hard to find possible biological interactions among them. To find possible mechanisms for the independent RNAs in the transcriptomic signature, it is wise to explore possible correlations between the independent RNAs and the remaining RNAs of CESC transcriptome. Thus, the transcriptomic signature could just provide the initial molecules rather than complete biological mechanisms for further experimental exploration.

For the protective RNAs in the transcriptomic signature, hsa-miR-532-5p was shown to be involved in many cancers either as a tumor suppressor or an oncogenic-miRNA (Song et al., 2015; Zhang J. et al., 2018). However, the role of hsa-miR-532-5p in CESC remains unknown, and our results prompted that it may exert a tumor suppressor role in CESC due to its positive correlation with OS of CESC patients. RNA-binding protein 38 (RBM38) was originally recognized as an oncogene and it was frequently found to be amplified in prostate, ovarian and colorectal cancer, chronic lymphocytic leukemia, colon carcinoma, esophageal cancer, dog lymphomas and breast cancer (Ding et al., 2015). But recently, more and more studies suggested that RBM38 might act as a tumor suppressor (Feldstein et al., 2012; Xue et al., 2014). Ding et al. found that the association between the expression of RBM38 and cancer prognosis varied from cancers and databases (Ding et al., 2015). These studies suggested that the function of RBM38 might be multidimensional in cancers. However, no study was performed to investigate the possible roles of RBM38 in CESC, and our analysis prompted to assume that RBM38 may be a tumor suppressor in CESC. Zic family member 2 (ZIC2) was shown to be oncogenic in many cancers such as ovarian cancer (Marchini et al., 2012) and hepatocellular carcinoma (Lu et al., 2017). In cervical cancer, ZIC2 was rarely investigated. Chan et al. (2011) demonstrated that ZIC2 was up-regulated in CC cell lines and the up-regulation of ZIC2 may enhance the activity of the Hedgehog signaling pathway through nuclear retention of Gli1. Although ZIC2 may be a risk factor that was deduced from the results of Chan et al., the prognostic role of ZIC2 in CESC patients was not investigated. Our analysis showed that ZIC2 may be a protective factor, and further studies are needed to elaborate on the disputable roles of ZIC2 in CESC.

Among the risky RNAs in the transcriptomic signature, Tyrosylprotein sulfotransferase 1 (TPST1) is an enzyme responsible for catalysis of tyrosine sulfation. Previous studies revealed that TPST1 could sulfate the tyrosine of C-X-C motif chemokine receptor (CXCR4) (Seibert et al., 2008; Xu et al., 2013) and the tyrosine sulfation might contribute to nasopharyngeal carcinoma metastasis (Xu et al., 2013). However, expressions, functions, and mechanisms of TPST1 in CESC are not clear. Consistent with these previous studies, our analysis revealed that TPST1 was harmful for CESC prognosis. C-X-C motif chemokine ligand 2 (CXCL2) was demonstrated to be up-regulated in many types of cancer such as chronic lymphocytic leukemia and bladder cancer. The up-regulation of CXCL2 could enhance the cell survival of lymphocytic leukemia (Burgess et al., 2012) and it was correlated with poor prognosis of bladder cancer (Zhang et al., 2016). Recently, Zhang et al. revealed that AKP1 could promote angiogenesis and tumor growth by up-regulating CXCL1, CXCL2, and CXCL8 in CC cells (Zhang W. et al., 2018). Our results revealed that CXCL2 was a risk factor for CESC prognosis, which is in line with these previous experimental studies in non-CESC cancers. Egl-9 family hypoxia inducible factor 1 (EGLN1) was a key cellular oxygen sensor, which played important roles in tumor angiogenesis (Chan and Giaccia, 2010) and tumor metastasis (Kuchnio et al., 2015). Both tumor-promoting and suppressive roles of EGLN1 have been reported in different types of cancer (Chan and Giaccia, 2010). Although EGLN1 was shown to be low-expressed in advanced CC (Roszak et al., 2011; Kuchnio et al., 2015), in our analysis, the expression of EGLN1 was a risk factor for CESC patient survival. Thus, further functional mechanisms of EGLN1 in CESC cells should be carried out to illustrate the worse prognostic effect of EGLN1. Myotubularin related protein 11 (MTMR11) was rarely reported in cancer. The lncRNA DLEU1 played multiple roles in different cancers. Previous studies revealed that DLEU1 could promote progression of ovarian carcinoma (Wang et al., 2017) and gastric cancer (Li et al., 2017a), while Balas and Johnson (2018). showed that DLEU1 may be a tumor suppressor. There are versatile forms of interaction for lncRNA. DLEU1 could exert its functions by binding to proteins (Li et al., 2017a; Liu et al., 2018) or miRNAs (Wang et al., 2017). Moreover, the up-regulated DLEU1 was shown to be associated with the survival of gastric cancer by promoting proliferation of gastric cancer cells (Li et al., 2017a). However, the potential roles of DLEU1 in CESC remain unclear, and our analysis revealed that DLEU1 may also exert as a tumor suppressor in CESC.

Some limitations of the current study should be noticed. (1) For PH assumption test, it is difficult to estimate the type II error (i.e., the false negative rate). Thus, it is hard to choose a threshold of PH assumption test *P*-value in multiple testing. (2) The transcriptomic signature was identified based on TCGA data-mining and further independent validations and mechanism explorations are in need. (3) Complete mechanisms could not be revealed by the transcriptomic signature itself. However, the transcriptomic signature may be of potential applications for clinical management of CESC patients. Specifically, we can measure the expressions of the transcriptomic signature in CESC patients and calculate the NPIs for the patients based on the measured expression values. Furthermore, CESC patients can be stratified as high-risk and low-risk based on their NPIs. Finally, for high-risk patients, more aggressive therapies such as high-dose chemoradiotherapy may be given. Moreover, hsa-miR-532-5p, RBM38, EGLN1, and DLEU1 were demonstrated as both oncogenes and tumor-suppressors in non-CESC cancers and their roles in CESC were unclear. This study provided experimental directions for these novel genes and miRNA.

## Conclusion

We have identified a novel transcriptomic signature for CESC prognosis, including 1 miRNA, 1 lncRNA and 6 mRNAs. The transcriptomic signature was more comprehensive and predictive than the miRNA-only, the gene-only, and the previously identified signatures for CESC prognosis.

## Author Contributions

JX conceived and designed the analysis. JX, SG, YS, LG, and ZB performed the analysis. JX wrote the paper.

## Conflict of Interest Statement

The authors declare that the research was conducted in the absence of any commercial or financial relationships that could be construed as a potential conflict of interest.

## Footnotes

1 https://cancergenome.nih.gov/

2 http://metascape.org

## Supplementary Material

FIGURE S1 Kaplan–Meier plots for age at diagnosis **(A)**, tobacco usage **(B)**, clinical stage **(C)**, and lymphovascular invasion **(D)**.

FIGURE S2 The first row shows the densities, the second the distribution function and the last row the local and tail area-based false discovery rates of miRNAs.

FIGURE S3 Schoenfeld residual plots for hsa-miR-335-3p and hsa-miR-532-5p.

FIGURE S4 The first row shows the densities, the second the distribution function and the last row the local and tail area-based false discovery rates of genes.

TABLE S1 MCA of RNA-NPI and miRNA-NPI.

TABLE S2 MCA of RNA-NPI and gene-NPI.

TABLE S3 MCA of the transcriptomic signature in non-mutated CESC samples.

TABLE S4 UCA of previously identified prognostic genes and miRNAs.

TABLE S5 MCA of previously indentified prognostic genes and miRNAs.

TABLE S6 MCA of RNA-NPI and pre-NPI.

METHODS Details on data retrieval and preprocessing.
