# Supplementary figures and images for: A Comprehensive RNA Expression Signature for Cervical Squamous Cell Carcinoma Prognosis
Source: Front Genet. 2019 Jan 4;9:696. doi: 10.3389/fgene.2018.00696 (PMC6328499; doi:10.3389/fgene.2018.00696)

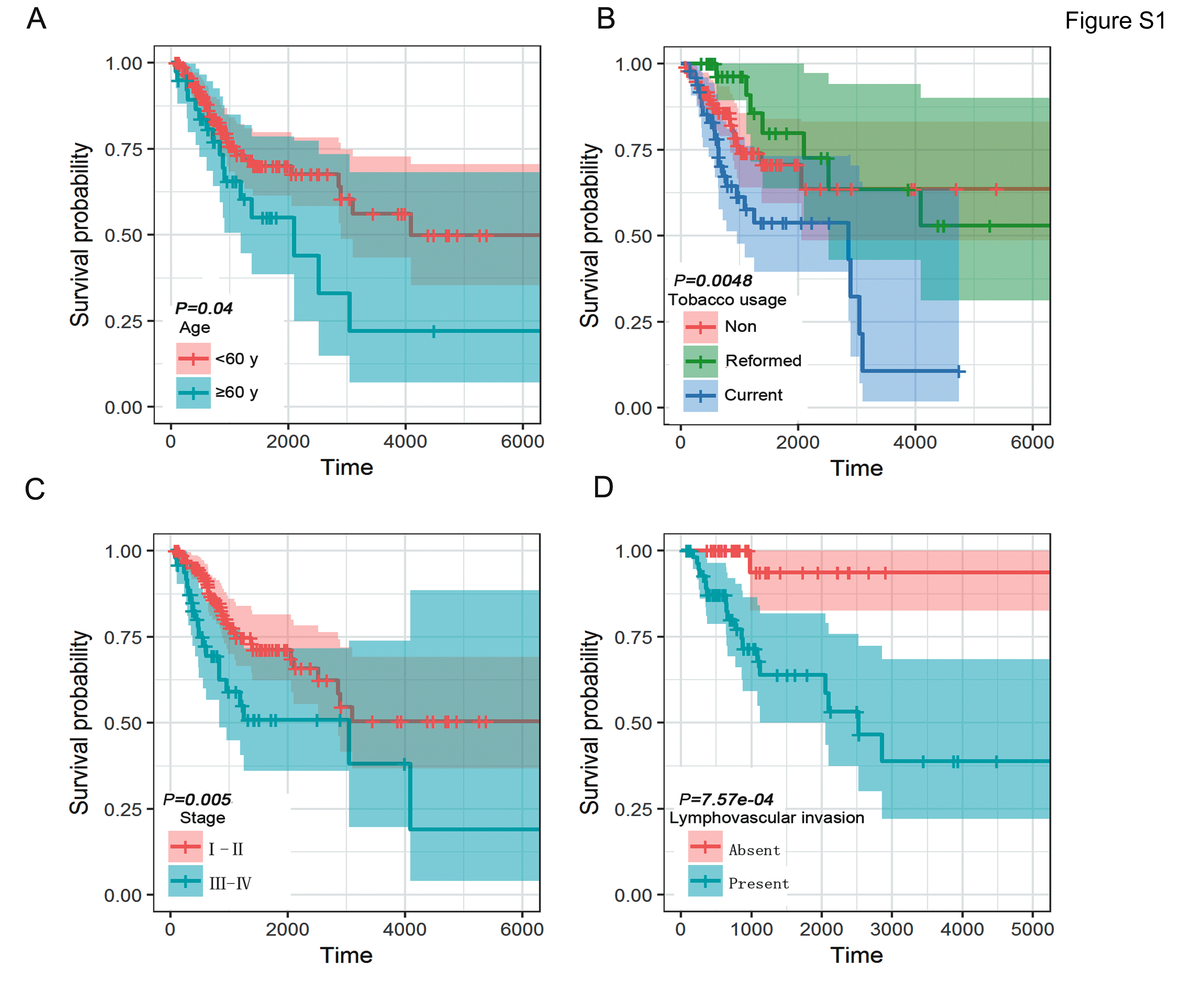

Supplement: FIGURE S1 — Kaplan–Meier plots for age at diagnosis (A), tobacco usage (B), clinical stage (C), and lymphovascular invasion (D). [file Image_1.TIF]

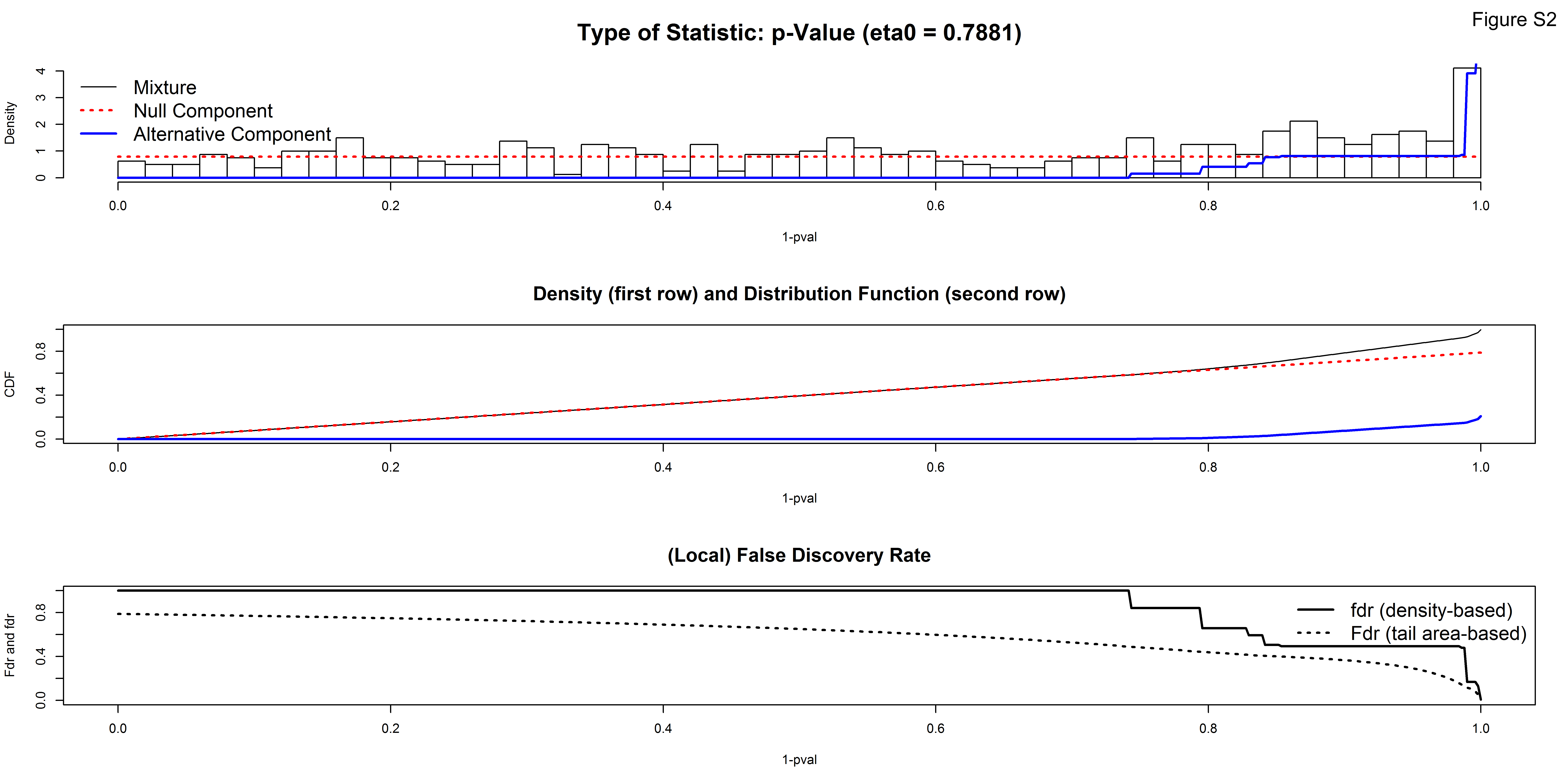

Supplement: FIGURE S2 — The first row shows the densities, the second the distribution function and the last row the local and tail area-based false discovery rates of miRNAs. [file Image_2.TIF]

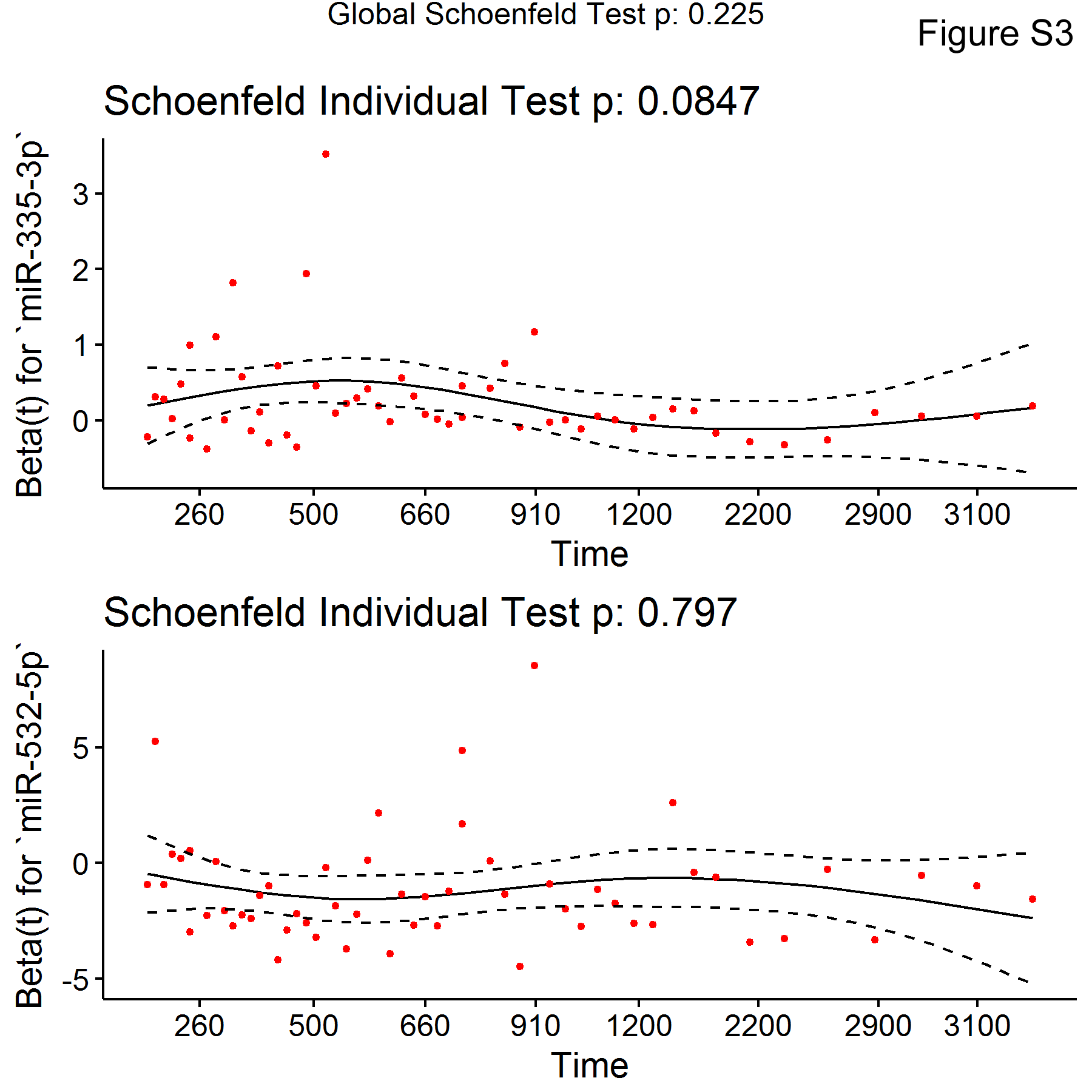

Supplement: FIGURE S3 — Schoenfeld residual plots for hsa-miR-335-3p and hsa-miR-532-5p. [file Image_3.TIF]

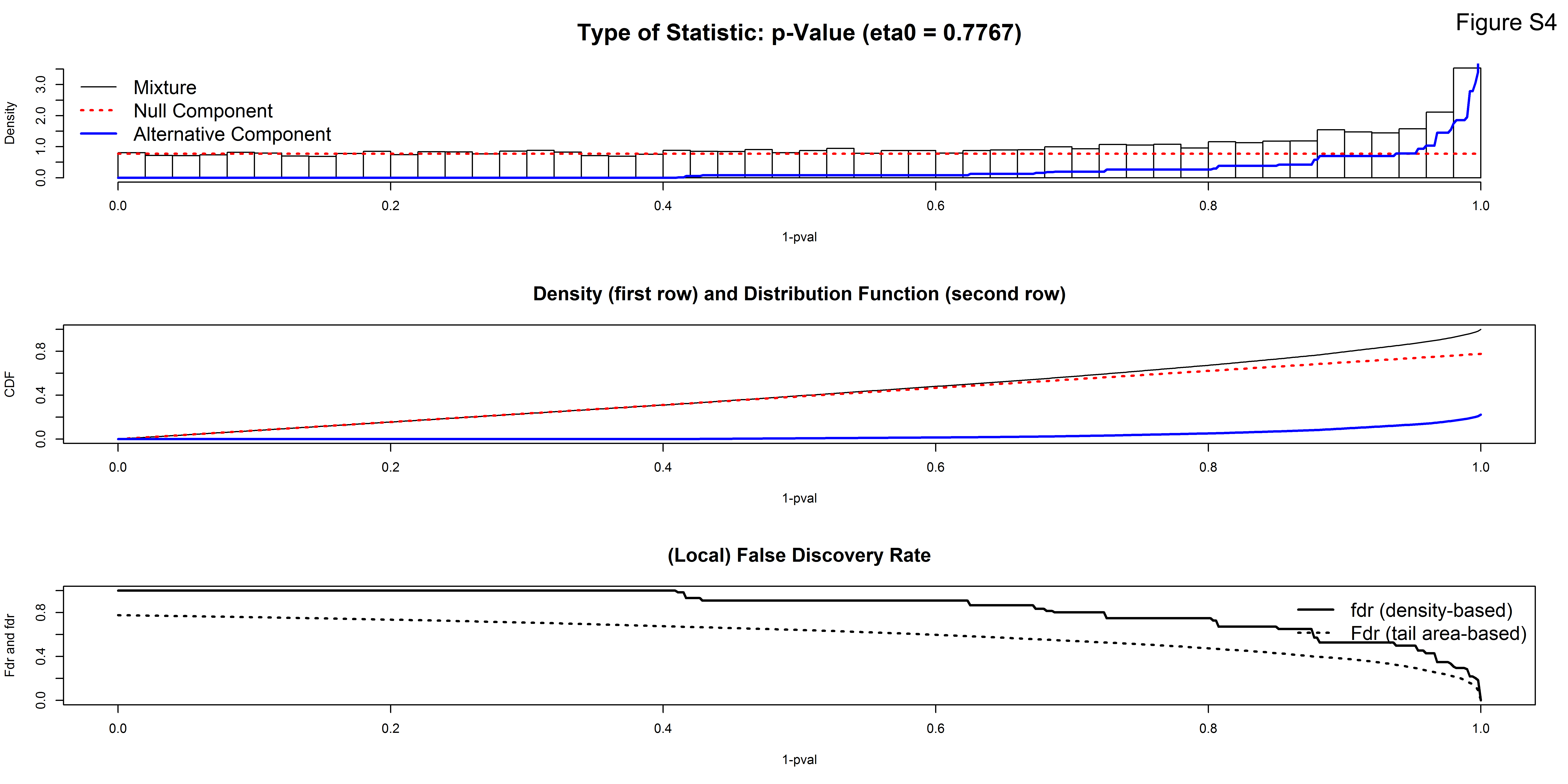

Supplement: FIGURE S4 — The first row shows the densities, the second the distribution function and the last row the local and tail area-based false discovery rates of genes. [file Image_4.TIF]
