# Supplementary material for: A Comprehensive RNA Expression Signature for Cervical Squamous Cell Carcinoma Prognosis
Source: Front Genet. 2019 Jan 4;9:696. doi: 10.3389/fgene.2018.00696 (PMC6328499; doi:10.3389/fgene.2018.00696)
Supplement: TABLE S1 — MCA of RNA-NPI and miRNA-NPI. [file Table_1.DOC]

**Supplementary material and methods**

**Section I**

***Data retrieval***

The search strategies are: (1) project = “TCGA-CESC”; (2) data category = “Clinical” and “Transcriptome Profiling” for clinical data and transcriptomic data, respectively; (3) data type = “Clinical supplement”, “Gene Expression Quantification”, and “Isoform Expression Quantification” for clinical data, gene expression data, and miRNA isoform expression data, respectively; (4) experimental strategy = “RNA-Seq” and “miRNA-Seq” for gene expression data and miRNA isoform expression data, respectively; and (5) workflow type = “HTseq-FPKM” and “GCGSC miRNA profiling” for gene expression data, and miRNA isoform expression data, respectively.

**Section II**

***Clinical data preprocessing***

The clinical eXtemsible Markup Language (XML) files were parsed by R “XML” package in to files that entitled “clinical_data.csv”, “durgs.csv”, “radiations.csv”, “new_tumor.csv”, and “follow_ups.csv”, respectively. The R code for clinical information parse is as follow.

#install.packages("XML")

#install.packages("stringr")

library(stringr)

library(XML)

library(methods)

#set working directory

clinical.manifest<-read.table(file="MANIFEST.txt",header=TRUE)#read your clinical file manifest

n.patient<-nrow(clinical.manifest)

filename<-as.character(clinical.manifest$filename)

clin<-list(clinical_data=NULL,follow_ups=NULL,new_tumors=NULL,drugs=NULL,radiations=NULL)

for (i in 1:n.patient){

xmlP<-xmlParse(filename[i], encoding="UTF-8")

Roots<-xmlRoot(xmlP)

xmlList<-xmlToList(xmlP)

value<-unlist(xmlList)

j<-value==""

value<-value[!j]

#****************clinical_information****************#

cut<-grep(pattern="new_tumor_events",x=names(value))

cut.point<-cut[1]-1

clinical_value<-value[1:cut.point]

j<-grepl(pattern="\\.text",names(clinical_value))

k<-grepl(pattern="\\.procurement_status",names(clinical_value))

j<-j|k

clinical_value<-clinical_value[j]

index.text<-grep(pattern="\\.text",x=names(clinical_value))

index<-grep(pattern="\\.procurement_status",x=names(clinical_value))

k<-(index-1)%in%index.text

clinical_value<-clinical_value[-index[k]]

j<-grepl(pattern="\\.text",names(clinical_value))

k<-grepl(pattern="\\.procurement_status",names(clinical_value))

names(clinical_value)[j]<-gsub(".text","",names(clinical_value)[j])

names(clinical_value)[k]<-gsub(".procurement_status","",names(clinical_value)[k])

n<-which(table(names(clinical_value))>1)

if(length(n)>0){

delet<-NULL

for(j in 1:length(n)){

k<-which(names(clinical_value)==names(n[j]))

a<-paste(clinical_value[k],collapse="&")

clinical_value[k[1]]<-a

delet<-c(delet,k[-1])

}

clinical_value<-clinical_value[-delet]

}

clin$clinical_data<-rbind(clin$clinical_data,clinical_value)

barcode<-clinical_value[which(names(clinical_value)=="patient.bcr_patient_barcode")]

#****************new_tumor_events****************#

new_tumor_value<-value[cut]

j<-grepl(pattern="\\.text",names(new_tumor_value))

k<-grepl(pattern="\\.procurement_status",names(new_tumor_value))

j<-j|k

new_tumor_value<-new_tumor_value[j]

index.text<-grep(pattern="\\.text",x=names(new_tumor_value))

index<-grep(pattern="\\.procurement_status",x=names(new_tumor_value))

k<-(index-1)%in%index.text

if(any(k==TRUE)){

new_tumor_value<-new_tumor_value[-index[k]]

k<-str_locate(string=names(new_tumor_value),pattern="new_tumor_events")

names(new_tumor_value)<-substr(names(new_tumor_value),k[,2]+2,sapply(names(new_tumor_value),nchar))

j<-grep(pattern="\\.text",names(new_tumor_value))

k<-grep(pattern="\\.procurement_status",names(new_tumor_value))

l<-str_locate(string=names(new_tumor_value)[j],pattern="\\.text")

m<-str_locate(string=names(new_tumor_value)[k],pattern="\\.procurement_status")

names(new_tumor_value)[j]<-substr(names(new_tumor_value)[j],1,l[,1]-1)

names(new_tumor_value)[k]<-substr(names(new_tumor_value)[k],1,m[,1]-1)

#barcode<-as.character(clin$clinical$patient.bcr_patient_barcode)

k<-which(names(new_tumor_value)=="new_tumor_dx_prior_submitted_specimen_dx")

a<-new_tumor_value[k]

new_tumor_value<-new_tumor_value[-k]

new<-names(new_tumor_value)

j<-which(new=="new_tumor_event_after_initial_treatment")

b<-new[-j]

len<-length(b)/max(table(b))

for(k in 1:(length(j))){

if(new_tumor_value[j[k]]=="YES"){

if(k!=length(j)){

n.new<-(j[k+1]-j[k]-1)/len

}else{

n.new<-(length(new_tumor_value)-j[k])/len

}

for(m in 1:n.new){

b<-new_tumor_value[((j[k]+1)+(m-1)*len):(j[k]+(m)*len)]

text<-c(patient.barcode=barcode,new_tumor_value[j[k]],a,b)

clin$new_tumors<-rbind(clin$new_tumors,text)

}

}else{

c<-rep("Not Available",len)

names(c)<-names(new_tumor_value)[(j[k]+1):(j[k]+len)]

text<-c(patient.barcode=barcode,new_tumor_value[j[k]],a,c)

clin$new_tumors<-rbind(clin$new_tumors,text)

}

}

}

#****************follow_ups****************#

value<-value[-cut]

cut<-grep(pattern="follow_ups",x=names(value))

if(length(cut)>0){

follow_ups_value<-value[cut]

value<-value[-cut]

j<-grepl(pattern="\\.text",names(follow_ups_value))

k<-grepl(pattern="\\.procurement_status",names(follow_ups_value))

j<-j|k

follow_ups_value<-follow_ups_value[j]

index.text<-grep(pattern="\\.text",x=names(follow_ups_value))

index<-grep(pattern="\\.procurement_status",x=names(follow_ups_value))

k<-(index-1)%in%index.text

follow_ups_value<-follow_ups_value[-index[k]]

j<-grep(pattern="\\.text",names(follow_ups_value))

k<-grep(pattern="\\.procurement_status",names(follow_ups_value))

l<-str_locate(string=names(follow_ups_value)[j],pattern="\\.text")

m<-str_locate(string=names(follow_ups_value)[k],pattern="\\.procurement_status")

names(follow_ups_value)[j]<-substr(names(follow_ups_value)[j],1,l[,1]-1)

names(follow_ups_value)[k]<-substr(names(follow_ups_value)[k],1,m[,1]-1)

#barcode<-as.character(clin$clinical$patient.bcr_patient_barcode)

n.follow<-max(table(names(follow_ups_value)))

len<-length(follow_ups_value)/n.follow

for(j in 1:n.follow){

text<-c(patient.barcode=barcode,follow_ups_value[((j-1)*len+1):(j*len)])

clin$follow_ups<-rbind(clin$follow_ups,text)

}

}

#****************drugs****************#

cut<-grep(pattern="drugs",x=names(value))

if(length(cut)>0){

drugs_value<-value[cut]

#value<-value[-cut]

j<-grepl(pattern="\\.text",names(drugs_value))

k<-grepl(pattern="\\.procurement_status",names(drugs_value))

j<-j|k

drugs_value<-drugs_value[j]

index.text<-grep(pattern="\\.text",x=names(drugs_value))

index<-grep(pattern="\\.procurement_status",x=names(drugs_value))

k<-(index-1)%in%index.text

drugs_value<-drugs_value[-index[k]]

j<-grep(pattern="\\.text",names(drugs_value))

k<-grep(pattern="\\.procurement_status",names(drugs_value))

l<-str_locate(string=names(drugs_value)[j],pattern="\\.text")

m<-str_locate(string=names(drugs_value)[k],pattern="\\.procurement_status")

names(drugs_value)[j]<-substr(names(drugs_value)[j],1,l[,1]-1)

names(drugs_value)[k]<-substr(names(drugs_value)[k],1,m[,1]-1)

#barcode<-as.character(clin$clinical$patient.bcr_patient_barcode)

if(length(drugs_value)>23&length(drugs_value)<46){

n.drugs=1

n<-which(table(names(drugs_value))>1)

delet<-NULL

for(j in 1:length(n)){

k<-which(names(drugs_value)==names(n[j]))

a<-paste(drugs_value[k],collapse="&")

drugs_value[k[1]]<-a

delet<-c(delet,k[-1])

}

drugs_value<-drugs_value[-delet]

}else{

n.drugs<-max(table(names(drugs_value)))

len<-length(drugs_value)/n.drugs

for(j in 1:n.drugs){

text<-c(patient.barcode=barcode,drugs_value[((j-1)*len+1):(j*len)])

clin$drugs<-rbind(clin$drugs,text)

#f<-c(f,barcode)

#z<-c(z,length(text))

}

}

}

#****************radiations****************#

cut<-grep(pattern="radiations",x=names(value))

if(length(cut)>0){

radiations_value<-value[cut]

value<-value[-cut]

j<-grepl(pattern="\\.text",names(radiations_value))

k<-grepl(pattern="\\.procurement_status",names(radiations_value))

j<-j|k

radiations_value<-radiations_value[j]

index.text<-grep(pattern="\\.text",x=names(radiations_value))

index<-grep(pattern="\\.procurement_status",x=names(radiations_value))

k<-(index-1)%in%index.text

radiations_value<-radiations_value[-index[k]]

j<-grep(pattern="\\.text",names(radiations_value))

k<-grep(pattern="\\.procurement_status",names(radiations_value))

l<-str_locate(string=names(radiations_value)[j],pattern="\\.text")

m<-str_locate(string=names(radiations_value)[k],pattern="\\.procurement_status")

names(radiations_value)[j]<-substr(names(radiations_value)[j],1,l[,1]-1)

names(radiations_value)[k]<-substr(names(radiations_value)[k],1,m[,1]-1)

#barcode<-as.character(clin$clinical$patient.bcr_patient_barcode)

n.radiations<-max(table(names(radiations_value)))

len<-length(radiations_value)/n.radiations

for(j in 1:n.radiations){

text<-c(patient.barcode=barcode,radiations_value[((j-1)*len+1):(j*len)])

clin$radiations<-rbind(clin$radiations,text)

}

}

}

**Section III**

***MiRNA and RNA sequencing data preprocessing***

MiRNA isoforms that only annotated as “mature” were considered to be eligible and those which annotated as “precursor”, “stemloop”, and “unannotated” were filtered out. The RNA sequencing data normalized by Fragments Per Kilobase Million were transformed into Transcripts Per Million. Finally, TCGA sample barcodes were matched by linking “md5” records that incorporated in “MANIFEST.txt” and “metadata*.JSON” files. RNAs that have at least one count per million expression in at least 80% CESC samples were retained and further scaled to zero mean and standard deviation.
