# Supplementary material for: A Comprehensive RNA Expression Signature for Cervical Squamous Cell Carcinoma Prognosis
Source: Front Genet. 2019 Jan 4;9:696. doi: 10.3389/fgene.2018.00696 (PMC6328499; doi:10.3389/fgene.2018.00696)
Supplement: TABLE S2 — MCA of RNA-NPI and gene-NPI. [file Table_2.docx]

Table S2. MCA of RNA-NPI and gene-NPI

| NPI | ^M^HR (95% CI) | ^M^P value |
| --- | --- | --- |
| RNA-NPI | 6.17 (1.82-20.88) | 0.003 |
| gene-NPI | 2.09 (0.77-5.63) | 0.147 |
