# Supplementary material for: A Comprehensive RNA Expression Signature for Cervical Squamous Cell Carcinoma Prognosis
Source: Front Genet. 2019 Jan 4;9:696. doi: 10.3389/fgene.2018.00696 (PMC6328499; doi:10.3389/fgene.2018.00696)
Supplement: TABLE S3 — MCA of the transcriptomic signature in non-mutated CESC samples. [file Table_3.docx]

Table S3. MCA of the transcriptomic signature in non-mutated CESC samples

| Signature | Symbol | ^M^HR (95% CI) | ^M^P value | type | Model P value |
| --- | --- | --- | --- | --- | --- |
| MIMAT0002888 | hsa-mir-532-5p | 0.30 (0.15-0.61) | 7.08e-04 | Protective | 8.449e-14 |
| ENSG00000132819.15 | RBM38 | 0.36 (0.22-0.58) | 4.23e-05 | Protective |  |
| ENSG00000081041.8 | CXCL2 | 1.65 (1.34-2.03) | 1.82e-06 | Risky |  |
| ENSG00000043355.9 | ZIC2 | 0.31 (0.18-0.54) | 3.00e-05 | Protective |  |
| ENSG00000014914.18 | MTMR11 | 1.58 (1.24-2.02) | 2.41e-04 | Risky |  |
| ENSG00000176124.10 | DLEU1 | 2.19 (1.60-3.00) | 9.84e-07 | Risky |  |
| ENSG00000135766.8 | EGLN1 | 1.77 (1.43-2.18) | 8.65e-08 | Risky |  |
| ENSG00000169902.12 | TPST1 | 1.32 (1.13-1.56) | 6.19e-04 | Risky |  |
