# Supplementary material for: A Comprehensive RNA Expression Signature for Cervical Squamous Cell Carcinoma Prognosis
Source: Front Genet. 2019 Jan 4;9:696. doi: 10.3389/fgene.2018.00696 (PMC6328499; doi:10.3389/fgene.2018.00696)
Supplement: TABLE S4 — UCA of previously identified prognostic genes and miRNAs. [file Table_4.docx]

Table S4. UCA of previously identified prognostic genes and miRNAs.

| Study | miRNA/gene | Isoform in TCGA | ^U^HR (95% CI) | ^U^P value | ^U^PHA test P value | Model P value |
| --- | --- | --- | --- | --- | --- | --- |
| Hu et al. [6] | miR-200a | miR-200a-5p | 0.718(0.497-1.037) | 7.72E-02 | 5.78E-01 |  |
|  |  | **miR-200a-3p** | **0.679(0.462-0.999)** | **4.92E-02** | **6.04E-01** |  |
|  | miR-9 | miR-9-1-5p | 0.751(0.520-1.084) | 1.27E-01 | 1.74E-01 |  |
|  |  | miR-9-2-5p | 0.752(0.521-1.086) | 1.28E-01 | 1.79E-01 |  |
|  |  | miR-9-3-5p | 0.752(0.521-1.085) | 1.28E-01 | 1.74E-01 |  |
|  |  | miR-9-3-3p | 0.919(0.706-1.195) | 5.28E-01 | 4.47E-01 |  |
| How et al. [7] | miR-let-7c | miR-let-7c-5p | 0.992(0.730-1.350) | 9.62E-01 | 1.74E-01 |  |
|  |  | miR-let-7c-3p | 0.985(0.748-1.296) | 9.12E-01 | 6.75E-01 |  |
|  | miR-21 | miR-21-5p | 1.109(0.854-1.441) | 4.39E-01 | 6.17E-01 |  |
|  |  | miR-21-3p | 0.917(0.691-1.216) | 5.48E-01 | 5.49E-02 |  |
|  | miR-222 | miR-222-5p | 1.005(0.760-1.329) | 9.74E-01 | 3.61E-01 |  |
|  |  | miR-222-3p | 1.063(0.820-1.377) | 6.45E-01 | 4.24E-01 |  |
|  | miR-451 | miR-451a | 1.206(0.953-1.526) | 1.19E-01 | 4.46E-01 |  |
|  | miR-455-5p | miR-455-5p | 0.892(0.681-1.167) | 4.03E-01 | 6.50E-01 |  |
|  | miR-134 | miR-134-5p | 1.012(0.775-1.321) | 9.29E-01 | 2.01E-01 |  |
|  | miR-148a | miR-148a-5p | 0.752(0.542-1.043) | 8.78E-02 | 3.75E-01 |  |
|  |  | miR-148a-3p | 0.755(0.552-1.031) | 7.73E-02 | 2.60E-01 |  |
|  | miR-218 | miR-218-1-5p | 0.779(0.546-1.112) | 1.69E-01 | 9.84E-01 |  |
|  |  | miR-218-2-5p | 0.806(0.581-1.118) | 1.96E-01 | 8.82E-01 |  |
|  | miR-500 | miR-500a-5p | 0.656(0.416-1.034) | 6.96E-02 | 1.48E-01 |  |
|  |  | **miR-500a-3p** | **0.374(0.176-0.795)** | **1.06E-02** | **1.68E-01** |  |
|  |  | miR-500b-5p | 0.659(0.419-1.038) | 7.18E-02 | 1.43E-01 |  |
| Liu et al. [8] | miR-142 | **miR-142-5p** | **0.660(0.480-0.906)** | **1.01E-02** | **9.91E-02** |  |
|  |  | **miR-142-3p** | **0.442(0.276-0.708)** | **6.90E-04** | **8.42E-02** |  |
|  | miR-642a | - | - | - | - |  |
|  | miR-101-1 | miR-101-1-5p | 0.760(0.551-1.048) | 9.42E-02 | 2.67E-01 |  |
|  |  | **miR-101-1-3p** | **0.575(0.409-0.807)** | **1.39E-03** | **7.52E-02** |  |
|  | miR-3607 | **miR-3607-3p** | **0.529(0.355-0.788)** | **1.72E-03** | **8.88E-01** |  |
|  | miR-502 | **miR-502-3p** | **0.383(0.243-0.604)** | **3.68E-05** | **1.53E-01** |  |
|  | miR-378c | **miR-378c** | **0.643(0.449-0.920)** | **1.56E-02** | **5.74E-02** |  |
|  | miR-150 | miR-150-5p | 0.615(0.436-0.869) | 5.78E-03 | 6.19E-03 |  |
| Liang et al. [9] | miR-145 | miR-145-5p | 0.727(0.509-1.038) | 7.93E-02 | 2.71E-01 |  |
|  |  | miR-145-3p | 0.859(0.650-1.137) | 2.88E-01 | 1.53E-01 |  |
|  | miR-200c | miR-200c-5p | 1.025(0.792-1.328) | 8.50E-01 | 8.90E-02 |  |
|  |  | miR-200c-3p | 1.066(0.858-1.324) | 5.64E-01 | 1.09E-01 |  |
|  | miR-218-1 | miR-218-1-5p | 0.779(0.546-1.112) | 1.69E-01 | 9.84E-01 |  |
| Zeng et al. [10] | miR-3154 | - | - | - | - |  |
|  | miR-7-3 | - | - | - | - |  |
|  | miR-600 | - | - | - | - |  |
| Ma et al. [11] | miR-642a | - | - | - | - |  |
|  | miR-378c | **miR-378c** | **0.643(0.449-0.920)** | **1.56E-02** | **5.74E-02** |  |
| Huang et al. [12] | UBL3 | ENSG00000122042 | 0.983(0.747-1.292) | 9.00E-01 | 2.29E-02 |  |
|  | FGF3 | ENSG00000186895 | - | - | - |  |
|  | BMI1 | ENSG00000168283 | 1.071(0.833-1.376) | 5.94E-01 | 7.92E-01 |  |
|  | PDGFRA | ENSG00000134853 | 1.144(0.942-1.389) | 1.76E-01 | 4.54E-01 |  |
|  | PTPRF | ENSG00000142949 | 1.063(0.839-1.347) | 6.13E-01 | 4.17E-01 |  |
|  | RFC4 | ENSG00000163918 | 0.803(0.589-1.095) | 1.65E-01 | 8.46E-01 |  |
|  | NOL7 | ENSG00000225921 | 0.901(0.668-1.214) | 4.92E-01 | 4.43E-01 |  |
| Li et al. [13, 14] | HIST1H2BD | **ENSG00000158373** | **0.676(0.472-0.969)** | **3.32E-02** | **7.43E-01** |  |
|  | HIST1H2BJ | **ENSG00000124635** | **0.271(0.113-0.647)** | **3.29E-03** | **3.93E-01** |  |
|  | MCM5 | **ENSG00000100297** | **0.663(0.481-0.913)** | **1.20E-02** | **7.33E-01** |  |

^U^, univariate analysis; PHA, proportional hazards assumption.
