# Supplementary material for: A Comprehensive RNA Expression Signature for Cervical Squamous Cell Carcinoma Prognosis
Source: Front Genet. 2019 Jan 4;9:696. doi: 10.3389/fgene.2018.00696 (PMC6328499; doi:10.3389/fgene.2018.00696)
Supplement: TABLE S5 — MCA of previously indentified prognostic genes and miRNAs. [file Table_5.docx]

Table S5. MCA of previously indentified prognostic genes and miRNAs.

| RNA | Symbol | ^M^HR (95% CI) | ^M^P value | type | ^M^Model P value |
| --- | --- | --- | --- | --- | --- |
| MIMAT0000434 | hsa-mir-142-3p | 0.56 (0.34-0.93) | 0.024 | Protective | 1.007e-07 |
| MIMAT0000099 | hsa-mir-101-1-3p | 0.59 (0.40-0.86) | 0.006 | Protective |  |
| MIMAT0004775 | hsa-mir-502-3p | 0.61 (0.38-0.96) | 0.033 | Protective |  |
| ENSG00000124635.8 | [HIST1H2BJ](http://asia.ensembl.org/homo_sapiens/Gene/Summary?g=ENSG00000124635&db=core) | 0.22 (0.09-0.56) | 0.001 | Protective |  |
