# Supplementary material for: A Comprehensive RNA Expression Signature for Cervical Squamous Cell Carcinoma Prognosis
Source: Front Genet. 2019 Jan 4;9:696. doi: 10.3389/fgene.2018.00696 (PMC6328499; doi:10.3389/fgene.2018.00696)
Supplement: TABLE S6 — MCA of RNA-NPI and pre-NPI. [file Table_6.docx]

Table S6. MCA of RNA-NPI and pre-NPI

| NPI | ^M^HR (95% CI) | ^M^P value |
| --- | --- | --- |
| RNA-NPI | 10.72 (5.98-19.21) | 1.55e-15 |
| pre-NPI | 2.46 (1.32-4.60) | 4.86e-03 |
